# Following the COVID-19 playbook and battling another infodemic: conspiracy beliefs around human monkeypox among the Lebanese population

**DOI:** 10.1186/s40545-023-00580-x

**Published:** 2023-06-13

**Authors:** Dalal Youssef, Lea Bleibel, Edmond Abboud

**Affiliations:** 1 Clinical Trials Program, Ministry of Public Health, Beirut, Lebanon; 2 Institut de Santé Publique d’Épidémiologie et de Développement (ISPED), Bordeaux University, Bordeaux, France; 3 Institut National de Santé Publique, Epidémiologie Clinique et Toxicologie (INSPECT-LB), Beirut, Lebanon; 4 Lebanese Higher Institute of Technical and Professional (IPNET), Beirut, Lebanon; 5 Notre Dame Jamhour, Baabda, Lebanon; 6 Ministry of Public Health, Beirut, Lebanon

**Keywords:** Conspiracy beliefs, Attitude, Monkeypox, COVID-19 playbook, Lebanon

## Abstract

**Introduction:**

The non-endemic multicountry outbreak of monkeypox (MPX) has emphasized the issue of conspiracy theories that go viral in times of societal crisis. Now, it is the turn of MPX to join COVID-19 in the conspiracy theory realm. Social media outlets were flooded by a scourge of misinformation as soon as MPX cases began to appear with an evident cross-pollination between diverse conspiracy theories. Given the adverse consequences of conspiracy beliefs, this study aimed to assess the extent of endorsement of MPX conspiracy beliefs among the Lebanese population and to identify its associated factors.

**Methods:**

Using a convenience sampling technique, a web-based cross section was conducted among Lebanese adults. Data were collected using an Arabic self-reported questionnaire. Multivariable logistic regression was performed to identify the factors associated with the MPX conspiracy beliefs scale.

**Results:**

Conspiracy beliefs regarding emerging viruses including MPX were detected among 59.1% of Lebanese adults. Participants endorsed particularly the conspiracy theories linking the virus to a deliberate attempt to reduce the size of the global population (59.6%), gain political control (56.6%) or pharmaceutical companies' financial gain (39.3%), in addition to the manmade origin of MPX (47.5%). Remarkably, the majority of surveyed adults exhibited a negative attitude toward the government's preparedness for a potential MPX outbreak. However, a positive attitude was revealed toward the effectiveness of precautionary measures (69.6%). Female participants and those having a good health status were less likely to exhibit a higher level of conspiracy beliefs. On the contrary, divorced or widowed adults, those having a low economic situation, poor knowledge level, and negative attitude either toward the government or precautionary measures were more prone to disclose a higher level of conspiracy beliefs. Notably, participants relying on social media to get information about MPX were also more likely to have a higher level of conspiracy beliefs compared to their counterparts.

**Conclusions:**

The widespread extent of conspiracy beliefs endorsement regarding MPX among the Lebanese population urged the policymakers to find ways to reduce people’s reliance on these theories. Future studies exploring the harmful impacts of conspiracy beliefs on health behaviors are recommended.

**Supplementary Information:**

The online version contains supplementary material available at 10.1186/s40545-023-00580-x.

## Background

Conspiracy theories emerged as attempts to explain the eventual causes of substantial social, political, or health-related events and circumstances with claims to identify hidden patterns and the culprits behind events by influencers [1]. These theories tried to render the inexplicable explicable and often appeal to people who are uncomfortable with uncertainty and randomness, especially during a frightening time. Previous studies have demonstrated that during societal crises, conspiracy theories spread-like wildfire [2, 3]. The conspiracy realm appears to be an empty shell of sorts that aggregates. Of note, a conspiracy theory is distinct from a rumor which is a story of the unknown with suspicion validity as well as from an authentic conspiracy which is an actual causal chain of events [4]. The conspiracy theories grasp essentially their power from their believers and supporters [5]. Thus, powerful and influential actors called conspirators were not essentially individuals who owned a real sociopolitical authority; they could be even powerless people, such as ethnic minorities. Apart from the identity of conspirators, malicious intent is veiled behind these theories [6]. The conspiracy theories encompassed sets of ready-made narratives that can be useful in different events and situations, and that is why intersecting and repetitive statements are noticed.

Several reasons could explain the reliance and the adherence of people to conspiracy theories [1, 7–9]. People may engage in conspiracy theories when epistemic, existential, and social needs are not fulfilled [3]. Epistemic need referred to people’s motivation to maintain certainty, consistency, and accuracy in their understanding of the world [10, 11]. The term “existential need” is used to describe people's need to feel safe, secure, and in control of their environment [12], while social need referred to people's drive to uphold a favorable and positive social image of themselves and the community to which they belong [13].

Notably, conspiracy theories pervaded several fields. For example, in the political world, the incorporation of conspiracy theories into propaganda was frequently used. The medical field was no exception and the belief in conspiratorial ideas is widespread [14]. Of note, the era of COVID-19 has created a sort of conspiracy playbook [15] that could serve as ingredients for any conspiracy theory that could be effectively and easily applied to other infectious diseases. This playbook is intended to spread misinformation and doubts, delegitimize public health officials and institutions, and stoke fear and vaccine hesitancy [16]. The ongoing multicountry outbreak of monkeypox (MPX) in non-endemic countries brought to focus the issue of conspiracy theories regarding emerging virus infections [17] and COVID-19 has found a new contemporary companion in the health-related conspiracy theory realm. Following the COVID-19 playbook, MPX conspiracy theories started spreading online almost as soon as cases began to appear outside of sub-Saharan Africa [18–20] and social media outlets were hotbed by misinformation. Although several prevalent MPX-related conspiracy theories are evolving constantly, an evident cross-pollination between diverse conspiracy theories was revealed [21]. This common phenomenon was owing to the self-perpetuating network of beliefs, where narratives often overlapped and copied each other [22]. Notably, it was anticipated that these narratives could ultimately mature into a single overarching conspiracy theory [19].

Along with the rise of MPX cases in different countries, the scourge of disinformation about them is to boot [23]. However, conspiratorial beliefs regarding MPX are not a novel phenomenon as similar concerns have been reported previously in endemic regions for the virus [24]. For example, a previous study in the Republic of the Congo reported the endorsement of false notions. This included beliefs that the virus was deliberately introduced into the area and even disbelief in the existence of the disease [24]. Currently, the main MPX conspiracies focus on the timing of the outbreak, the virus’ real origin, and the usual “who profits from it?” issue. One of the conspiracy theories was related to the prophecy of the Simpsons, a 33-year-old cartoon, about the MPX outbreak. Before the MPX outbreak, this cartoon was claimed to foresee some events, such as the COVID-19 pandemic [25]. Tweets and Facebook posts showing misleading images went viral [26]. Similar to COVID-19, MPX was regarded as a hoax created by a global cabal. It was also alleged that MPX could have political routes as well as it could be human-made and intentionally deliberated [27]. Other rumors that lacked any evidence, connected the emergence of MPX to COVID-19 vaccines [28]. In addition, MPX is being weaponized to attack black and Lesbian, gay, bisexual, transgender, queer, and ally (LGBTQ +) communities across the world [29]. It should be noted that some racial, homophobic, and transphobic tinges always backed these kinds of conspiracy theories [30]. Similar to previous disease outbreaks (e.g., Zika, COVID-19, and Ebola), conspiracy theories have also focused on pharmaceutical companies’ role in exaggerating the severity of MPX for financial and political gains and the marketing of MPX vaccines [18].

However, the normalization and mainstreaming of conspiracy theories represent a big concern [31]. This is a trend that is not going away so easily, and it will likely continue to evolve. The latter was driven by several factors, such as the rise of social media and the growing media polarization [32, 33]. In addition, these theories are also very useful, because they are cheap and effective political weapons.

Of note, conspiracy beliefs could have significant concerns for the prevention, treatment, and aftermath of disease outbreaks [34, 35], such as decreasing compliance with disease-prevention measures [36, 37] as well as increasing people’s reluctance to vaccination [37, 38]. To date, no study in Lebanon tackled this subject despite the widespread conspiracy beliefs in the country during the COVID-19 pandemic and the vaccine hesitancy encountered during the national roll-out of the COVID-19 vaccination plan. Therefore, it is important to explore the extent of belief in emerging virus conspiracy theories among the Lebanese population. Understanding conspiracy beliefs and their underlying mechanisms are of great interest because of their relationship with both non-preventive behaviors and refusal of vaccination and also with government attitudes. Thus, even comparatively small numbers of individuals who endorse conspiracy beliefs could undermine health-related efforts. This study will provide an overall picture of the conspiracy beliefs' popularity toward emerging diseases among the Lebanese population and offers a unique perspective about its associated factors to orient interventions that aim to reduce people’s reliance on conspiracy theories and, therefore, limit their harmful effects.

The overarching objective of this study was to assess the extent of endorsement of conspiracy beliefs regarding emerging diseases with a special focus on MPX and to identify its associated factors.

## Materials and methods

### Study design

As part of a larger project focusing on the knowledge, attitudes, and beliefs of the Lebanese population toward MPX, a web-based cross-sectional survey was conducted during the first 2 weeks of August 2022 among Lebanese adults. All Lebanese adults aged 18 years or above, willing to read and understand the Arabic language, and living currently in Lebanon, were eligible to participate in this study. Given its online nature, this study excluded adults who lacked internet literacy and those who did not have access to internet service at the time of the study. In addition, non-Lebanese adults, Lebanese adults living outside Lebanon, and those who refused to participate were also excluded from the study. A convenience sampling method was used to recruit participants from all Lebanese governorates (Bekaa, Beirut, Mount Lebanon, Baalbeck Hermel, South, Nabtyeh, North, and Akkar).

### Questionnaire development

A self-administered, questionnaire was initially developed in English. The questionnaire was designed to cover MPX knowledge among Lebanese adults as well as their attitudes toward precautionary measures, country preparedness, and their beliefs regarding conspiracy theories. The internal consistency reliability of the English version of both knowledge (*α* = 0.88) and attitudes sores (precautionary measures (*α* = 0.73), country preparedness (*α* = 0.92), conspiracy beliefs (*α* = 0.81) was estimated using Cronbach’s alpha, where its value *α* ≥ 0.70 was considered satisfactory [39]. To assess the content validity and to confirm whether the tool adequately comprises all the items necessary to cover the study objective, an expert panel composed of eight members (two epidemiologists, two infectious disease specialists, two psychologists, and two lay experts) was appointed [40]. Experts were defined as individuals who had a good understanding of the MPX and were aware of the MPX-related conspiracy theories. Experts assessed the relevancy or representativeness and clarity of the items to measure the construct operationally‏ defined by these items [41]. Based on the content validity index (CVI) calculated both at the item level (I-CVI) and scale level (S-CVI) (CVI greater than or equal to 0.78), the panel of experts judged that the questionnaire had good content validity [42].

Based on the standard translation guidelines [43], the original English draft of the questionnaire was translated and adapted to the Arabic language. Any suggested change was resolved by consensus. The reliability of the Arabic version of the questionnaire was also confirmed. A pre-test of the questionnaire was performed among 30 Lebanese adults from different Lebanese governorates to ensure the readability, clarity, and comprehensibility of the questions as well as the survey flow. Based on the feedback of the participants in the pre-test, minor changes were made to the questionnaire including the replacement of some ambiguous words. The average time for completing the survey was 15 min.

The questionnaire consisted of three main sections and open-ended questions:

- **The baseline characteristics of the study participants section** included the sociodemographic variables (age, gender, marital status, urbanicity, education level, and occupation of the participants. Participants were also asked to rank their health and economic status. They were also queried if they have been diagnosed with MPX and whether they know someone infected by the MPX.
- **The attitude section comprised the following three subsections:**
- Attitude toward MPX conspiracy beliefs: The items used by a study conducted by Freeman et al. [44]on coronavirus conspiracy beliefs were adopted and extended to cover the current MPX-related conspiracy theories and to assess the participants’ attitude toward conspiracy explanations of emerging virus infections, particularly the MPX. The assessment was performed using 16 items with a 3-Likert scale of possible responses (disagree [1], neutral [2], agree [3]. As previously mentioned, the content validity of the scale was assessed and The internal consistency of the score was ensured by calculating a Cronbach’s alpha value.

The following items comprised the emerging virus infections conspiracy scale (EVICS) adapted to the current MPX outbreak as well as four additional items. (1)“I am skeptical about the official explanation regarding the cause of MPX emergence”, (2) “I do not trust the information about the viruses including MPX from scientific experts”, (3) “Most viruses including MPX are man-made (e.g., NIT simulation)”, (4) “The spread of viruses including MPX is a deliberate attempt to reduce the size of the global population”, (5) “The spread of viruses including MPX is a deliberate attempt by governments to gain political control”, (6) “The spread of viruses including MPX is a deliberate attempt by global companies to take control including pharmaceutical companies manufacturing vaccines”, (7) “The spread of MPX is a deliberate attempt to attack African people and to enhance discrimination”, (8) “The spread of MPX is a deliberate discriminatory attempt to attack LGBTQ+”, (9)“The control measures including Lockdowns in response to emerging infection are aimed for mass surveillance and to control every aspect of our lives”, (10) “The control measures in response to emerging infection are aimed for mass surveillance and to destabilize the economy for financial gain , (11) “The control measures including lockdown is a way to terrify, isolate, and demoralize a society as a whole to reshape society to fit specific interests”, (12) “Viruses including MPX are biological weapons manufactured by the superpowers to take global control”, (13) “Viruses including MPX were a plot by globalists to destroy religion by banning gatherings”, (14) “The mainstream media is deliberately feeding us misinformation about the virus (MPX) and lockdown”, (15) “The COVID-19 vaccination is linked to the MPX outbreak” (16) “Microsoft co-founder (Bill Gates) have a role in the outbreak”. All attributed values were summed and the overall level would range from 16 to 48 points. Higher scores pointed to a higher level of conspiracy beliefs regarding MPX virus emergence and subsequent intervention measures. Of note, the conspiracy belief scale was categorized into two categories: low and high levels based on the scale median.

b. Attitude toward the country's preparedness and readiness to respond to a potential MPX outbreak: this subsection included 16 items covering different the adequacy of activities taken by Lebanon to prepare for a potential MPX outbreak (surveillance, preventive measures at point of entry, travel recommendations, testing, awareness) as well as their confidence and trust about the ability of the country to deal with a potential outbreak. Of note, these questions were developed based on the current Lebanese situation and took into consideration the multilayered crises. Responses to questions related to attitude were ranked on a 3-point Likert scale, an agreement scale ranging from "1" for the option "disagree" to "3" for the option "agree". A point of 1 was given to the respondent's answer that selected the "agree" option, while "disagree" or "neutral" responses were given a score of 0 points. Alike the knowledge score, the attitude score in each domain was categorized using the original Bloom's cutoff point, as positive if the score was 60–100% and negative if the score was less than 60%.
c. Lebanese adults’ attitudes toward the effectiveness of the recommended prevention measures against MPX: this subsection comprised the following items (1) isolation is an effective technique to prevent the spread of MPX (2) regular hand hygiene, physical distancing, and facemask use could protect people from catching MPX, (3) Keeping up with the information regarding the government’s call for MPX preventive efforts is important for the community and (4) People with MPX who isolate themselves show that they have a responsibility in preventing the transmission of COVID-19. Similarly, to the previous subsection, responses were answered using a 3-point Likert scale. This score in each domain was also categorized using the original Bloom's cutoff point.
d. **Knowledge about the MPX** **section:**

It consisted of 55 items covering different aspects of MPX knowledge (etiology, clinical presentation, severity, transmission routes, case management, vaccines, and precautionary measures). A detailed description of this section was already tackled by another manuscript focusing on the knowledge of MPX among the Lebanese population. Each question was answered on a true/false basis and with an additional “I don’t know” option. A value of ‘1′ was attributed to a correct response and a value of 0 was attributed to "wrong" or don’t know. All attributed values were summed and the overall level would range from 0 to 55 points. Using modified Bloom’s cutoff point, the overall knowledge level was categorized as good if the score was ≥ 60% (33–55 points), and poor if the score was less than 60% (< 33 points). Participants were also inquired about their sources of information regarding HPMX.

### Sample size calculation

Using the Raosoft sample size calculator, the required sample size was calculated. No previous study in Lebanon examined the population's belief in MPX conspiracy beliefs; a conservative estimate of 50% was used. Based on a 95% confidence level, and an absolute error of 5%, a minimum sample size of 383 was required. A larger sample was collected to reduce errors related to the sampling technique and to increase the study power, a rough estimation was made by multiplying the calculated sample size by 2 times, leading to a final sample size of 793 participants.

### Ethical considerations

Informed electronic consent was obtained from each participant before his enrolment in the study. The research protocol was properly reviewed and approved by the ethical committee at Rafic Hariri University Hospital (reference number 2022-0801). All methods were performed following the ethical standards as laid down in the Declaration of Helsinki and its later amendments. Participants were reassured that the participation is voluntary and that they were free to withdraw at any time. In addition, information was gathered anonymously and handled confidentially.

### Statistical analysis

The generated data on an excel spreadsheet were transferred to the statistical software IBM SPSS® software (Statistical Package for Social Sciences) version 24.0 for analysis. Descriptive statistics were reported using frequency with percentages for categorical variables. The responses to all questions were mandatory; therefore, no missing data to be a substitute. For descriptive analysis, frequency and percentage were used for categorical variables, and mean and standard deviation for quantitative variables. The normality distribution of knowledge scale items, conspiracy scale items, and each attitude items scale was confirmed by the calculation of skewness and kurtosis values which are lower than 1 [45]. The Chi-squared test was used to compare the means between 2 categorical variables. All variables that showed a *p* value < 0.2 in the bivariate analysis were included in the multivariable analysis as independent variables. Binary logistic regression using the stepwise method was conducted to identify the correlates of the conspiracy belief scale, after checking the absence of multi-collinearity. *P* values less than 0.05 were considered to be statistically significant for two-sided statistical tests.

## Results

### Baseline characteristics of the participants

A total of 793 Lebanese adults from all Lebanese governorates completed this study. Most of the participants were females and nearly half of them were married and aged between 18 and 30 years. Around the third quarter of them had a university level or above and were living in urban areas. More than 70% of them performed outside the healthcare sector. Of note, the majority of surveyed adults reported good health status (74.91%) and were not suffering from chronic diseases or immunodeficiency (80.71%). In terms of the economic situation, more than the third quarter ranked their current economic situation as low (77.56%).

### Attitudes of the Lebanese adults

#### Conspiracy beliefs toward MPX

After checking skewness and Kurtosis (skewness =  − 0.690, kurtosis = 0.923), the EVICS score was found normally distributed. The MPX conspiracy scale ranged between 16 and 48 with a score mean of *M* = 32.67, standard deviation (SD = 6.01), and median = 34. Regarding conspiracy scale items holding the highest level of agreement, 59.6% of the study respondents agreed that the spread of the MPX virus is a deliberate attempt to a deliberate attempt to reduce the size of the global population (59.6%). Similarly, 56.6% of them endorsed that the spread of MPX is a deliberate attempt by authorities to gain political control or by global companies to take control including pharmaceutical companies for financial gains and the marketing of MPX vaccines (39.3%). Around half of the Lebanese adults (47.5%) found that most emerging viruses including MPX are planned and man-made (NIT simulation). Of note, 34.7% of Lebanese adults were skeptical about the official explanation regarding the cause of MPX emergence and 33.0% of them do not trust the information given by scientific experts about MPX. As for considering viruses including MPX as biological weapons manufactured by the superpowers to take global control, only 20.9% of the study respondents agreed with this claim. Other MPX conspiracy theories were less believed by the Lebanese population such as those that are tinged with a discriminatory background. For example, a limited proportion of Lebanese believed that MPX is a deliberate discriminatory attempt to attack African people (9.7%) or LGBTQ +  (14.8%). Remarkably, only 2.1% of respondents linked the COVID-19 vaccine to the emergence of MPX (Table 1).

**Table 1:** Conspiracy beliefs around MPX among the Lebanese population

		Disagree	Neutral	Agree
		*n* (%)	*n* (%)	*n* (%)
C1	I am skeptical about the official explanation regarding the cause of MPX emergence”	72 (9.1)	446 (56.2)	**275 (34.7)**
C2	I do not trust the information about the MPX from scientific experts	176 (22.2)	355 (44.8)	**262 (33.0)**
C3	Most viruses including MPX are planned and man-made (NIT simulation)	54 (6.8)	362 (45.6)	**377 (47.5)**
C4	The spread of viruses including MPX is a deliberate attempt to reduce the size of the global population”,	89 (11.2)	231 (29.1)	**473 (59.6)**
C5	The spread of viruses including MPX virus is a deliberate attempt by authorities to gain political control”	63 (7.9)	281 (35.4)	**449 (56.6)**
C6	The spread of viruses including MPX is a deliberate attempt by global companies to take control including pharmaceutical companies manufacturing vaccines	50 (6.3)	431 (54.4)	**312 (39.3)**
C7	The spread of MPX is a deliberate attempt to attack African people and enhance discrimination	155 (19.5)	561 (70.7)	77 (9.7)
C8	The spread of MPX is a deliberate discriminatory attempt to attack LGBTQ +	217 (27.4)	459 (57.9)	117 (14.8)
C9	The control measures in response to emerging infection are aimed for mass surveillance and to control every aspect of our lives”,	177 (22.3)	539 (68.0)	77 (9.7)
C10	The control measures in response to emerging infections are aimed for mass surveillance and destabilizing the economy for financial gain	264 (33.3)	453 (57.1)	76 (9.6)
C11	The control measures including lockdown are a way to terrify, isolate, and demoralize a society as a whole to reshape society to fit specific interests	269 (33.9)	444 (56.0)	80 (10.1)
C12	Viruses including MPX are biological weapons manufactured by the superpowers to take global control	40 (5.0)	587 (74.0)	**166 (20.9)**
C13	MPX was a plot by globalists to destroy religion by banning gatherings”,	156 (19.7)	556 (70.1)	81 (10.2)
C14	The mainstream media is deliberately feeding us misinformation about the MPX and lockdown	82 (10.3%)	572 (72.1)	139 (17.5)
C15	The COVID-19 vaccination is linked to the MPX outbreak,	344 (43.4)	432 (54.5)	**17 (2.1)**
C16	Microsoft co-founder and billionaire Bill Gates has a role in the outbreak	233 (29.4)	44 (56.4)	113 (14.2)

#### Attitudes toward the preparedness and readiness of the country

**Fig. 1:**
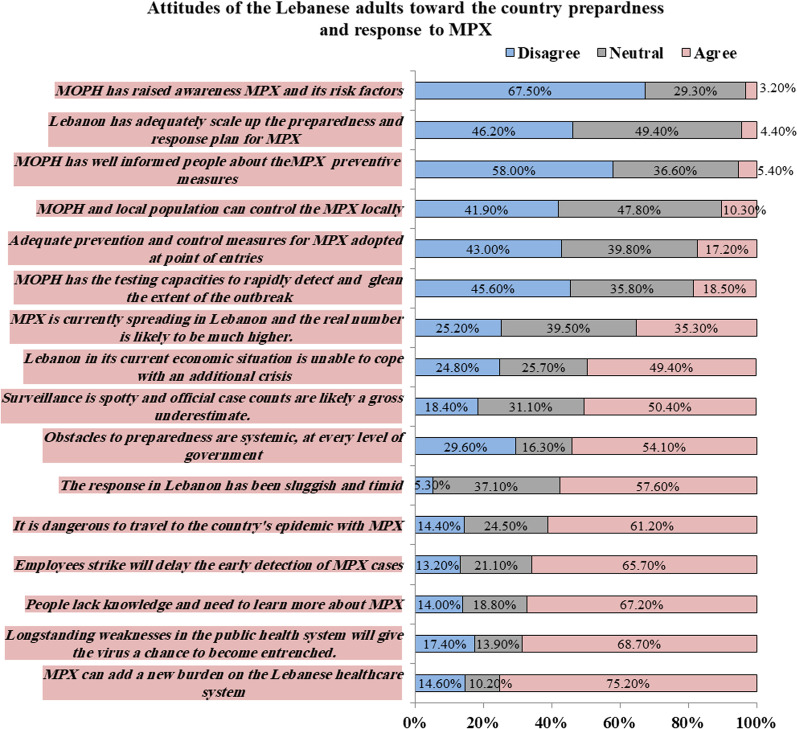
Attitudes of Lebanese adults toward the preparedness and readiness of the country

Briefly, 4.4% of the surveyed adults thought the country has adequately scaled up the preparedness and response plan for MPX and only 10.3% of them believed in the aptitude of the Lebanese population and the health authorities to control locally a potential outbreak. Around the third quarter (75.2%) of them believed that the MPX will add a burden to the Lebanese healthcare system which already suffered from a longstanding weakness and this will give the MPX virus a chance to become entrenched (68.7%). Furthermore, around half of them thought that Lebanon will be unable to cope with a potential outbreak of MPX due to its current economic crisis. They also alleged that employees' strikes will delay the detection of suspected MPX cases (65.7%) and that allowing people to travel to MPX epidemic countries is dangerous (61.2%). Around half of them thought that the Lebanese response to MPX was sluggish and timid (57.6%) and that obstacles to good preparedness were systemic and existed at each level of the government. In terms of MPX surveillance, half of the surveyed adults assumed that surveillance is spotty and 35% of them believed that MPX is currently spreading in Lebanon and the real number is likely to be much higher than the official case counts. As for laboratory testing capacities, only 18.5% agreed that Lebanon has the laboratory capacity to rapidly detect and glean the extent of the outbreak. In terms of MPX awareness, less than 5% of participants thought that MOPH has well-informed the public regarding MPX, its risk factors, and its specific precautions measures. Therefore, 67.20% of them assumed that Lebanese people lacked knowledge about MPX and need to learn more (Fig. 1).

#### Attitudes toward the effectiveness of the precautionary measures

**Fig. 2:**
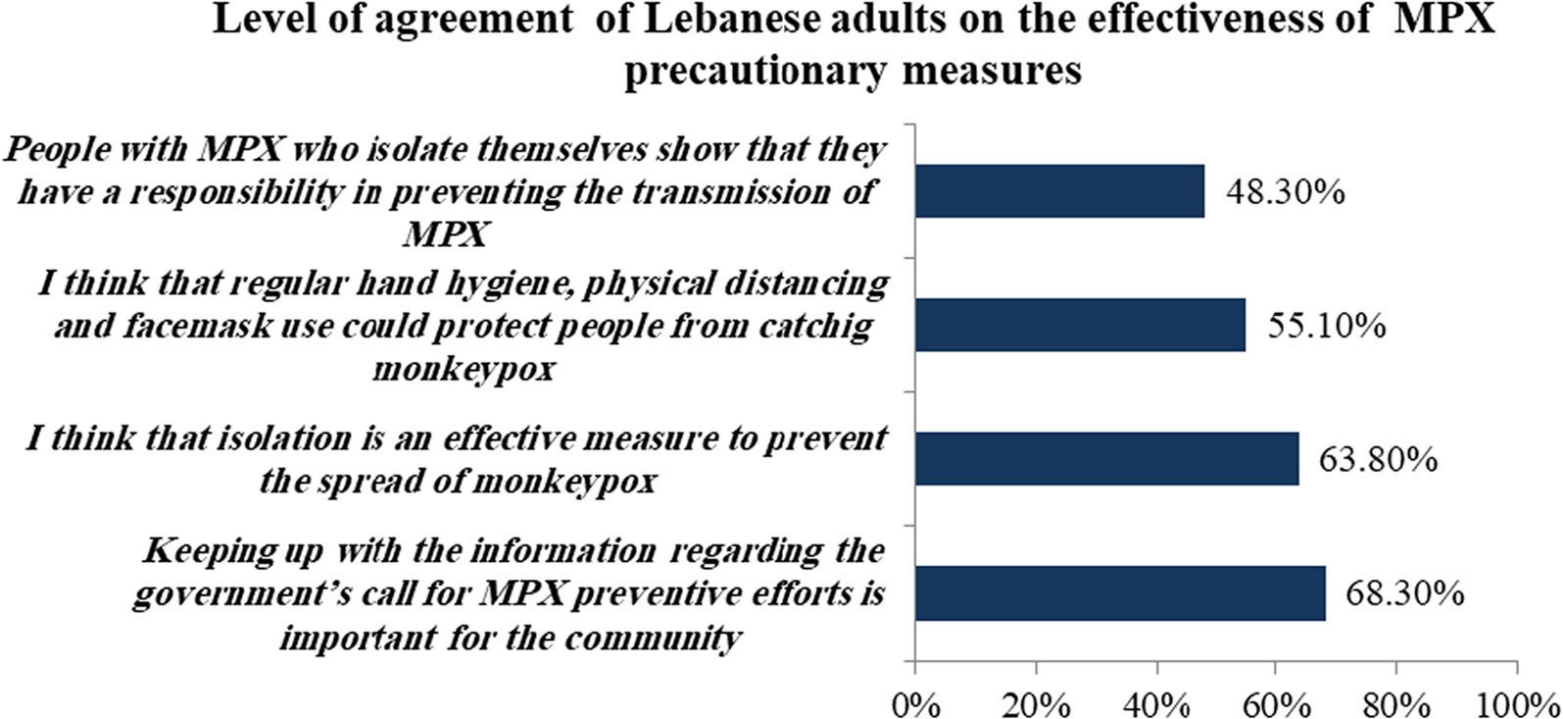
Level of agreement of Lebanese adults on the effectiveness of MPX precautionary measures

As shown in Fig. 2, 68.3% of participants agreed that keeping updated on the recommendations issued by the government regarding MPX preventive measures is essential for combating the disease. In addition, more than half of them believed that isolation is an effective measure to stop the spread of MPX (63.8%) as well as regular hand hygiene; physical distancing and facemask use could protect people from being infected with MPX (55.1%). Of note, 48.3% of them thought that people with MPX who isolate themselves show that they have a responsibility in preventing the transmission of MPX.

### Overall knowledge Level of MPX among the Lebanese population

**Fig. 3:**
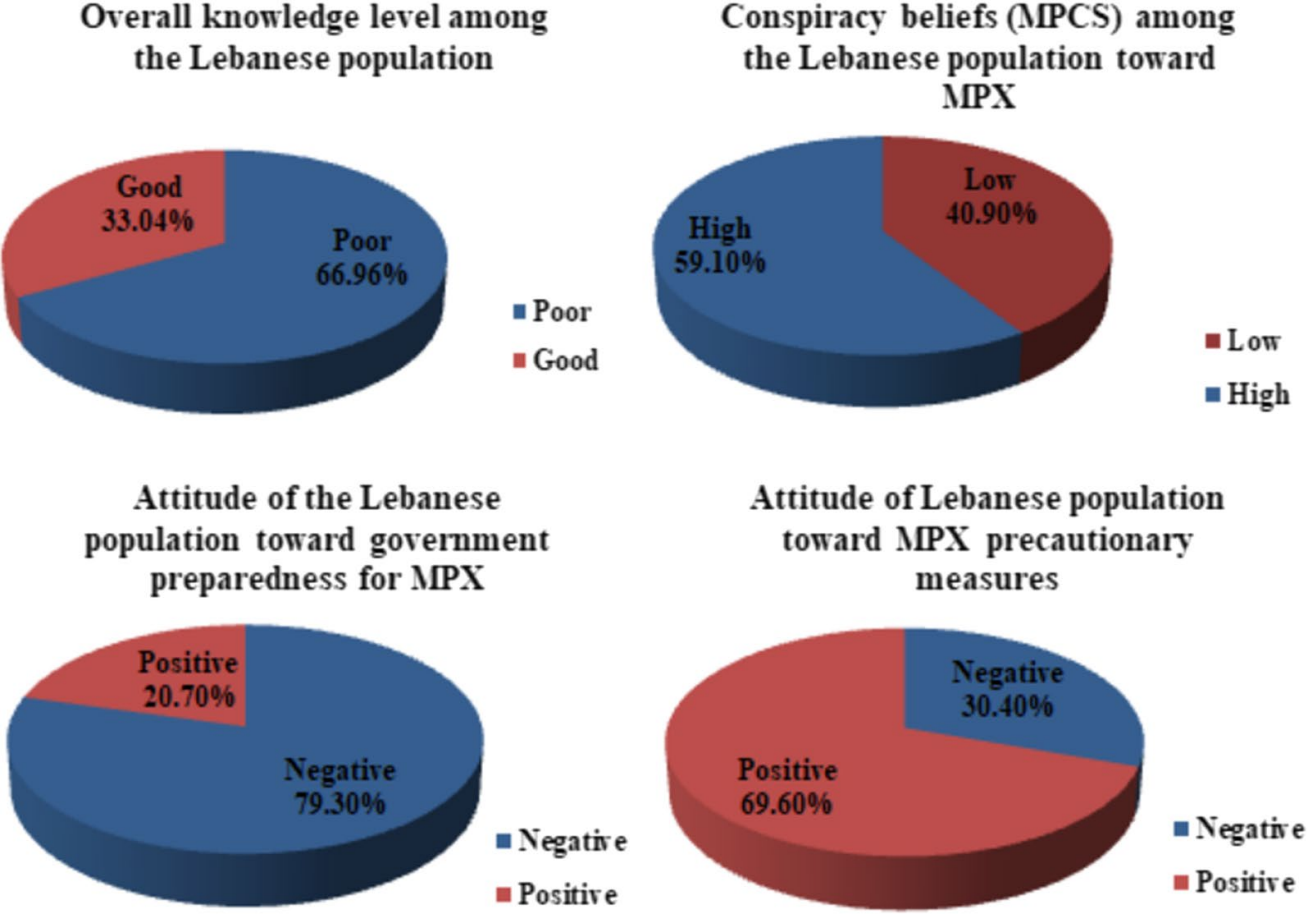
MPX attitudes, knowledge, and conspiracy belief scores

The majority of the Lebanese population (66.96%) had a poor level of knowledge regarding MPX (Fig. 3). Most of the participants referred to media, health authorities, and social media to get information about MPX.

### Attitudes scores

As shown in Fig. 3, the majority of surveyed adults (59.1%) had high conspiracy beliefs toward MPX, and more than the third quarter of them (79.3%) exhibited a negative attitude toward the government's preparedness for a potential MPX outbreak. On the other hand, the majority of them (69.6%) revealed a positive attitude toward MPX precautionary measures.

### Relationship between conspiracy beliefs, knowledge, attitude toward government, and attitude toward precautionary measures

A significant difference was found in terms of conspiracy beliefs between participants with a poor knowledge level regarding MPX and those with good knowledge. Poor knowledge was found significantly associated (*p* < 0.001) with higher conspiracy beliefs about MPX. A significant difference was also found between adults with a positive attitude and those with negative attitudes in terms of the level of endorsement of conspiracy theories. On the contrary, a positive attitude toward precautionary measures (*p* < 0.001) or government preparedness (*p* = 0.009) was associated with lower conspiracy beliefs (Table 2).

**Table 2:** Relationship between MPX conspiracy beliefs, knowledge score, and attitudes

	MPX Conspiracy beliefs		Total	*P* value
	Low	High		
	*n* (%)	*n* (%)	*n*	
Overall knowledge score				< 0.001
Good	79 (30.2)	183 (69.8)	262	
Poor	125 (23.6)	406 (76.4)	531	
Attitude toward precautionary measures				< 0.001
Negative	57 (23.65)	184 (76.35)	241	
Positive	267 (48.4)	285 (51.6)	552	
Attitude toward government preparedness				0.009
Negative	248 (39.4)	381 (60.6)	629	
Positive	76 (46.3)	88 (53.7)	164	

*N* frequency, *%* Percentage

### Relationship between conspiracy beliefs and sociodemographic variables

**Fig. 4:**
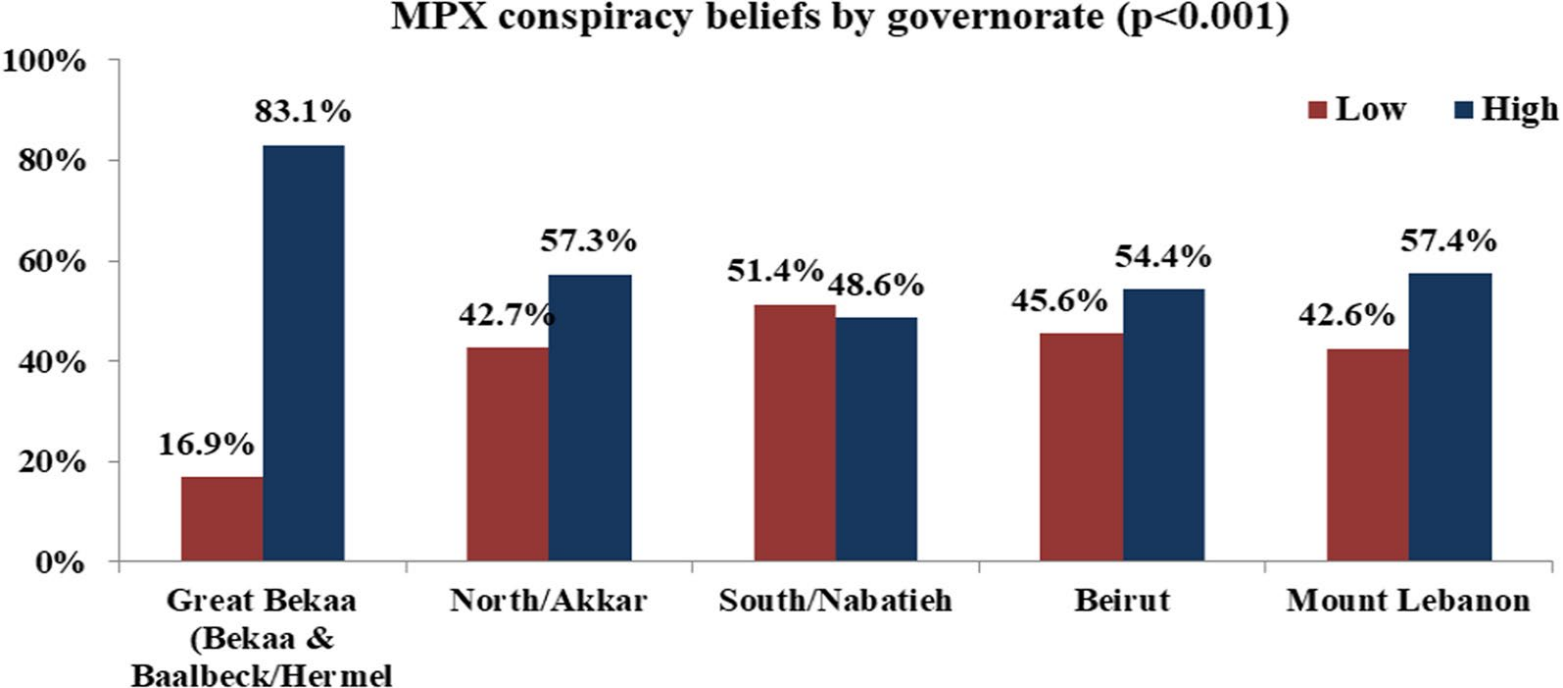
MPX conspiracy beliefs among Lebanese population by governorate

Table 3 shows the relationship between the knowledge score and the socio-demographics of respondents. A significant difference in terms of gender (*p* = 0.001), age groups (*p* < 0.001), marital status (*p* < 0.001), education (*p* < 0.001), health status (*p* < 0.001), and economic situation (*p* < 0.001). All these variables had a significant relationship with MPX conspiracy belief. Age was positively associated with MPX conspiracy belief score, which increased, with most participants (72.6%) aged 50 and above scoring high conspiracy belief (≥ 34). A gender difference was also revealed as females showed lower MPX conspiracy beliefs compared to male participants. A high level of MPX conspiracy beliefs was more likely to be found among divorced/widowed (86.4%) compared to married and single participants. A significant decrease level of MPX conspiracy belief was found among participants with higher education levels (56.3%) compared to those having a secondary level of education or below (67.9%). No difference was found in terms of urbanicity or occupation. There was also a significant variance between the MPX conspiracy score and the health status and economic situation. Participants with lower economic backgrounds and with fair health status reported a higher level of conspiracy beliefs. As shown in Fig. 4, a significant difference was also revealed between residency governorates, where participants living in Bekaa showed a higher MPX conspiracy beliefs level.

**Table 3:** Relationship between MPX conspiracy beliefs, sociodemographic, and health-related variables

	MPX conspiracy beliefs			
	Low	High	Total	*P* value
	*n* (%)	*n* (%)	*n*	
Gender				0.001
Male	74 (32%)	157 (68%)	231	
Female	250 (44.5%)	312 (55.5%)	562	
Age (years)				< 0.001
18–29	183 (51.7%)	171 (48.3%)	354	
30–49	89 (35.7%)	160 (64.3%)	249	
50 and above	52 (27.4%)	138 (72.6%)	190	
Marital status				< 0.001
Single/Married	316 (43.1%)	418 (56.9%)	734	
Widowed/divorced	8 (13.6%)	51 (86.4%)	59	
Educational level				< 0.001
Secondary or below	62 (32.1%)	131 (67.9%)	193	
University or above	262 (43.7%)	338 (56.3%)	600	
Occupation				0.712
Outside the medical field/not working	235 (40.9%)	339 (59.1%)	574	
Medical field	89 (40.6%)	130 (59.4%)	219	
Urbanicity				0.432
Urban	245 (41.7%)	343 (58.3%)	588	
Rural	79 (38.5%)	126 (61.5%)	205	
Overall health status				< 0.001
Fair or below	55 (27.6%)	144 (72.4%)	199	
Good or above	269 (45.3%)	325 (54.7%)	594	
Self-reported economic situation				< 0.001
Low	204 (33.1%)	411 (66.9%)	615	
Moderate/high	120 (67.4%)	58 (32.6%)	178	

*N* frequency, *%* Percentage

### Relationship between conspiracy beliefs and sources of information

A significant difference was revealed between MPX conspiracy beliefs and sources of information used to get information about MPX (Table 4). The majority of respondents who used social media (85.7%) reported higher conspiracy beliefs compared to those who did not use this source (21.8%) to get information (*p*<0.001). As for those relying on health authorities (*p*=0.048), health professionals (*p*<0.001), and health websites (*p*<0.001), they reported lower conspiracy beliefs compared to surveyed adults who did not use these sources. As for media (TV, radio, newspaper) and family or friends, no significant difference was revealed in terms of MPX conspiracy beliefs between users of these sources and those who did not use them.

**Table 4:** Conspiracy beliefs and sources of information

	Conspiracy beliefs			
	Low	High	Total	
	*n* (%)	*n* (%)	*n*	*P* value
Sources of information about MPX				
Media (TV, radio, newspaper)				0.131
No	115 (46.37)	133 (53.6)	248	
Yes	209 (38.35)	336 (61.7)	545	
Social media (WhatsApp, Facebook, Twitter..)				< 0.001
No	258 (78.2)	72 (21.8)	330	
Yes	66 (14.25)	397 (85.7)	463	
Health authorities (MOPH…)				0.048
No	75 (24.3)	234 (75.7)	309	
Yes	249 (51.44)	235 (48.56)	484	
Healthcare professionals (physicians…)				< 0.001
No	89 (19.14)	376 (80.86)	465	
Yes	235 (71.6)	93 (28.4)	328	
Scientific journals				0.238
No	83 (22.25)	290 (77.74)	373	
Yes	241 (57.38)	179 (42.62)	420	
Health website (WHO, CDC…)				< 0.001
No	106 (28.57)	265 (71.14)	371	
Yes	218 (51.65)	204 (48.34)	422	
Family and friends				0.324
No	148 (33.4)	295 (66.6)	443	
Yes	176 (50.3)	174 (49.7)	350	

*n* frequency, *%* percentage

### Factors associated with MPX conspiracy beliefs

High conspiracy beliefs level was found negatively associated with female gender, geographical area, and good health status. Female participants were less likely to have a higher embrace of conspiracy beliefs compared to males (aOR = 0.769, CI 95% (0.527–0.921). In comparison with participants coming from Bekaa governorate, participants from other governorates such as Beirut (aOR = 0.339, CI 95% (0.174–0.662), Mount Lebanon, (aOR = 0.218, CI 95% (0.116–0.409), North and Akkar (aOR = 0.299), CI 95% (0.143–0.627), and South and Nabatieh (aOR = 0.207, CI 95% (0.103–0.415) were less likely to have higher level of conspiracy beliefs. Similarly, participants who reported good health status (aOR = 0.598, CI 95% (0.389–0.921) were less prone to endorse conspiracy beliefs compared to their counterparts. As for age, participants aged between 30 and 49 years (aOR = 1.367, CI 95% (1.197–2.609) and those aged 50 and above (aOR = 1.820, CI 95% (1.260–2.840) were more likely to have a higher conspiracy beliefs level compared to young participants aged between 18 and 29 years. Interestingly, being divorced or widowed (aOR = 4.703, CI 95% (2.109–7.488) was found associated with an increase in the likelihood of endorsing conspiracy beliefs by 4.7 times. Similarly, participants with a low economic situation (aOR = 2.312, CI 95% (1.488–3.411) were more likely to have higher conspiracy beliefs compared to those with a moderate/high economic situation. In regards to knowledge, participants with poor knowledge levels (aOR = 2.826, CI 95% (1.983–4.027) were 2.8 times more prone to endorse conspiracy beliefs compared to those who have a good knowledge score. As for attitudes, adults with negative attitudes either toward government preparedness (aOR = 2.599, CI 95% (2.268–4.760) or toward the effectiveness of precautions measures (aOR = 3.286, CI 95% (2.268–4.760) were more likely to display a higher level of conspiracy beliefs. Finally, participants relying on social media to get information about MPX were 3.2 times more likely to endorse conspiracy theories (aOR = 3.294, CI 95% (3.582–7.824) compared to those who do not use social media for this purpose (Table 5).

**Table 5:** Factors associated with MPX conspiracy beliefs

	*P* value	aOR	CI 95%	
			Lower	Upper
Gender	0.002			
Male	Ref.			
Female		0.769	0.527	0.921
Age (years)	**0.016**			
18–29	Ref.			
30–49		1.367	1.197	2.609
50 and above		1.820	1.260	2.840
Marital status	** < 0.001**			
Single/Married	Ref.			
Widowed/divorced		4.703	2.109	7.488
Governorate	< 0.001			
Great Bekaa (Bekaa and Baalbeck/Hermel	Ref.			
North/Akkar		0.299	0.143	0.627
South/Nabatieh		0.207	0.103	0.415
Beirut		0.339	0.174	0.662
Mount Lebanon		0.218	0.116	0.409
Overall health status	** < 0.001**			
Fair or below				
Good or above		0.598	0.389	0.921
Self-reported Economic situation	** < 0.001**			
Moderate/high	Ref.			
Low		2.312	1.488	3.411
Overall knowledge level	< 0.001			
Good	Ref.			
Poor		2.826	1.983	4.027
Attitude toward precautionary measures	< 0.001			
Positive				
Negative		3.286	2.268	4.760
Attitude toward government				
Positive				
Negative		2.599	1.983	5.431
Source of information: social media				
No	Ref.			
Yes		3.294	3.582	7.824

*aOR* adjusted odds ratio, *CI* Confidence interval

### Discussion

Conspiracy theories go viral in times of societal crisis [2, 14] and the belief in health conspiracy theories is prevalent [14]. The COVID-19 playbook developed during the pandemic has served as a primary material for any infectious disease conspiracy theory and now, it is the turn of MPX to face the same destiny as COVID-19. Based on data from a representative survey of the Lebanese population, this study is the first to explore the extent of belief in emerging virus conspiracy theories with a special focus on MPX among the Lebanese population and determine and correlate knowledge of the MPX, sociodemographic variables and sources of information with conspiracy beliefs. It provides a backdrop of conspiracy beliefs in Lebanon and could be helpful to prepare for a potential outbreak and combatting concomitant infodemic by reducing people’s reliance on conspiracy theories and thus limiting their harmful consequences especially since the public seems vulnerable to social media outlets afflicted by a scourge of misinformation. Of note, the results of this study, conducted at the national level, corroborate and extend those of existing research at the international level.

## Main findings

A high level of conspiracy beliefs regarding emerging virus infections including MPX was found among 59.1% of the surveyed adults. The extent to which people embrace these theories varies significantly across the various geographical regions, socio-demographic characteristics (age, gender, marital status) as well as their knowledge levels and attitudes toward government and precautionary measures. In addition, social media was implicated in increasing conspiracy beliefs among the population.

In terms of conspiracy beliefs, the wide prevalence of endorsing such beliefs among the Lebanese population regarding MPX is expected especially in the era of pandemics. A previous study showed that believing in health-related conspiracy theories is universal [14]. In addition, emerging diseases including COVID-19 and MPX could be an almost ideal breeding ground for these beliefs [20]. Our results were consistent with the findings of a study conducted among Jordanian university students who also exhibited a high level of conspiracy beliefs despite their higher educational level [46]. One possible explanation for the large extent of endorsement of conspiracy theories among Lebanese people could be related to their inability to gain control over the multilayered crises encountered by the country in the real world. Previous research reported that conspiracy thinking is common, especially during high times of economic, political, and social crisis [47]. Hence, the overlapping emergencies in the country including the ongoing economic and political crises, as well as the strained public health system, could explain to a certain extent the temptation of Lebanese adults to fall back on conspiracy theories to rationalize the unexpected. For that reason, they may try to restructure an illusion to adjust to their stressful life events [48]. In addition to the lack of sense of control, the information and knowledge vacuum revealed in a previous study regarding MPX could trigger such conspiracy thinking. Humprecht et al. (2020) theorized that a country’s resilience to conspiracy theories depends on several media systems, political, and economic indicators [49]. Finally, such incline among the Lebanese adults to believe in conspiracy theories and misinformation should not be marginalized or regarded as a fringe phenomenon with a minor impact on real-world engagements, as several studies conducted across different countries demonstrate the detrimental consequences of these beliefs on the self, others, and society at large [34, 50, 51]. The latter could affect essentially the prevention, treatment, and aftermath of disease outbreaks [34, 51] such as compliance with disease-prevention measures [36, 52] and vaccine hesitancy [37, 38].

In regards to the highest endorsed conspiracy theories, participants embrace particularly those linking the virus to a deliberate attempt to reduce the size of the global population, gain political control or pharmaceutical companies' financial gain, in addition to the manmade origin of MPX (47.5%). Similar to previous disease outbreaks (e.g., Zika, COVID-19, and Ebola), conspiracy theories focus on pharmaceutical companies’ role in exaggerating the severity of MPX for financial and political gains [18] and the marketing of MPX vaccines. It was alleged also that this was human-made and intentionally deliberated. Of note, these conspiracy theories were copied from the COVID-19 playbook which is not surprising, since MPX re-emergence coincided with the COVID-19 pandemic and both are viral infections.

Findings from this study indicated also a significantly low agreement about the government preparedness and response toward a potential MPX outbreak and this negative attitude was found to lead to a higher conspiracy belief level. These findings could be understood in light of the current Lebanese situation as the country navigates dark times and the population struggle amid economic collapse and political instability [53]. The emerging diseases (COVID-19, MPX…) have added fuel to the fire. In addition, the country faces a growing shortage of medical supplies and essential medicines leaving the most vulnerable people at risk [54]. Therefore, one possible explanation could be the disgust of the Lebanese population toward the political system that increases epistemic mistrust toward government preparedness and the tendency to believe in conspiracy theories. Our results were consistent with the findings of a previous study regarding the association between disease-related conspiracy theories and lower levels of trust in governmental and health institutions [55]. The unveiled negative attitude toward government preparedness brought attention to the important role of the Government and health authorities in communicating risk and involving mass media in developing the beliefs of the community about the government’s actions and boosting people's trust toward the adequacy of these measures.

On the other hand, this study revealed a positive attitude toward the effectiveness of precautionary measures among the majority of Lebanese adults. Such a finding could be attributed to the good knowledge level of this domain found by Youssef et al. in a recent study conducted among the Lebanese population [56]. Of note, earlier studies [57, 58] suggested that the success of national response strategies in fighting emerging diseases and the increase of the community's compliance with preventive measures [59] depends on this community's attitudes toward the importance of preventive measures. In other words, these attitudes play a key role in inducing people's self-protection behaviors [60, 61]. Furthermore, the study findings meaningfully extend previous research on the link between conspiracy theories and attitudes toward precautionary measures in the context of emerging diseases, where a high level of conspiracy beliefs was found among people who exhibited a negative attitude toward precautionary measures. These results were in line with the findings of a previous which found that disease-related conspiracy beliefs are associated with less willingness to follow restrictive measures to inhibit the further spread of the disease [52].

In terms of socio-demographics, some interesting findings came to light. First, age was significantly associated with conspiracy beliefs: older respondents believed more strongly in these narratives than the younger generations. On the contrary, other studies emphasized that young people are more inclined to embrace such beliefs [62, 63]. Lebanese older individuals were with virtually no national welfare system and were stranded amid their country’s worsening economic catastrophe. In their prime, they survived years of civil war, economic crises, and bouts of instability. Many of them are now living in poverty as a result of one of the world's greatest financial crises in the previous 150 years. Therefore, older people who felt powerless were more prone to believe emerging disease-related conspiracy theories. Although that older generations, tend to consume slightly less social media, a study showed that people affected by historical traumas tend to interpret current events using conspiratorial frameworks developed during the traumatic event “traumatic rift” [64]. Therefore, older adults represented an important target group to be addressed specifically when planning information or prevention campaigns using more easily accessible and understandable information about the outbreak.

Second, gender was a significant factor in believing in conspiracy theories. This study showed that males were more likely to believe in conspiracy theories than females. However, no clear pattern of gender differences in endorsing conspiracy theories was revealed in previous studies and its results are mixed. For example, some studies reported that women are more likely to consider COVID-19 conspiracy theories [65–67], others found no gender differences [44, 68], while some found that men are more likely to endorse COVID-19 conspiracy theories [69] which are in line with our results.

Interestingly, marital status was significantly associated with a higher level of emerging disease conspiracy beliefs. Although some studies did not find any association between marital status and conspiracy beliefs [70], this study showed that divorced/widowed adults were more prone to believe in conspiracy beliefs than married or single adults. One possible explanation could be that beliefs in conspiracy theories are indeed such remnants of human adaptation to historical traumas and the high level of beliefs in these theories is related to personality characteristics, antecedents, and a sense of powerlessness [71, 72]. As for divorce, it could be psychologically traumatic, because if unexpected, the individual could feel shocked and powerless by the event. Hence, the divorcee could also feel pain, confusion, and deep, emotional scarring. As for the widowed, the death of the partner constituted by itself a traumatic event. In addition, loneliness and the inability to rely on a partner could also increase the embrace of conspiracy beliefs. A study showed that people who were less secure and more avoidant were among individuals endorsing the conspiracy item [73]. Given these results, it may be worth exploring more factors increasing higher conspiracy beliefs among this group.

One peculiar finding was that adults living in the Bekaa governorate exhibited a higher level of conspiracy beliefs compared to participants from other governorates. It should be noted that geographical social structures that shape citizens’ feelings of vulnerability and powerlessness could predict conspiracy beliefs [74]. In general, people from Bekaa were considered conservative and studies showed that conservative people are more likely to endorse conspiracy theories [75]. In addition, belief in conspiracy theories is highly sensitive to social context. A deep understanding of Bekaa’s environment and the drivers of such a higher level of conspiracy beliefs among Bekaa people is recommended.

Inconsistently with previous research [1, 46, 76, 77], educational attainment was not found associated with the extent of endorsement of conspiracy beliefs among the Lebanese population. As for adults with good health status, they were found less likely to embrace emerging diseases conspiracy beliefs. This could be explained by the fact that this category feels safer and did not consider themselves at high risk of the disease.

Regarding the economic situation, this study showed that participants with low economic situations were more likely to embrace conspiracy beliefs. Previous studies revealed relationships between lower income [78] and a higher endorsement of conspiracy theories. In addition, people who perceive their economic situation as deteriorating tend to view the perpetrators as collectively hoarding resources.

In terms of knowledge level, participants with low knowledge levels were found more likely to endorse conspiracy beliefs. In line with our findings, a study assessing knowledge of MPX viral infection among the general population in Saudi Arabia found that myths believers had low MPX knowledge levels [79]. Likewise, a study conducted by Sallam et al. [46] showed that a high MPX knowledge score was associated with a lower embrace of conspiracy belief. One possible explanation is that knowledge emphasizes analytic thinking logically and provides rational explanations against conspiracy theories.

In line with the literature, this study showed that the use of social media to get information about MPX was associated with greater conspiracy beliefs level. Several studies reported that conspiracy thinking is associated with a tendency to acquire information through digital media which include the internet and social media [49, 80, 81]. However, this was an anticipated and not surprising phenomenon as digital media systems contain misinformation and panic at the same velocity [82] inside all the important late-breaking data. Therefore, social media has been considered a breeding ground for misinformation, such as conspiracy theories [80] which are repeated and perpetuated and this puts public health at a constant in the crosshairs. However, the negative side of social media also comes with a positive impact, hence is crucial to improve our systems understanding of the flow of information and do more to protect people from harmful content related to the outbreaks.

## Limitations

The results of the study should be interpreted in light of several limitations that included: the cross-sectional design of the study which does not allow us to infer causality, hence, its findings should be interpreted as correlational. Selection bias is possible due to the convenience technique that was used to collect data which limits the generalizability of the findings. However, our sample was large enough to decrease to some extent bias related to the sampling technique and to increase the study power. Some drawbacks related to the online nature of the study should be admitted such as the difficulty to obtain a truly random sample of participants as the participation is limited to those having internet service, active users, and those who are available at the time of the researchers post the instrument and start the data collection. In addition, the survey may only have been completed by those who were digitally literate and those who were sufficiently interested in the topic to take the time and trouble to respond (the participation was voluntary and no incentive was given for participation). Finally, this survey was distributed through social media platforms; therefore, there is no way of identifying, and describing the population that could have accessed and responded to the survey.

## Implications

While the world is fighting emerging diseases, it is also combating an infodemic in which falsehoods tend to spread faster than truths and evidence. Therefore, conspiracy beliefs should receive a lot of attention during health crises. The present findings add to the body of research on conspiracy beliefs by assessing the level of conspiracy and its associated factors. Our findings can also be helpful for better planning for a potential MPX outbreak especially since cases of MPX are still scarce in the community. Interventions emphasizing and boosting analytic thinking through the provision of logic and rational arguments against specific conspiracy theories should be developed by policy-makers to reduce the appeal of these theories among Lebanese adults. In addition, enhancing feelings of trust, security, and a sense of confidence among the public is also recommended. As we prepare for MPX, it is also important to deepen our understanding of how people think, feel, and behave during disease outbreaks in particular. Since there is still much-unexplored territory to be discovered in the psychology of conspiracy theories, forthcoming studies exploring the potential impacts of these theories on health behaviors that prevent the spread of infectious diseases were suggested as well as the human tendency to believe conspiracy theories. Furthermore, longitudinal studies assessing conspiracy beliefs at multiple timepoints (pre-outbreak, during the outbreak, and post-outbreak) are of great interest. Finally, disgust could be associated with people’s perception of moral violation in the system and make people suspect that the government is not telling to them the whole story about MPX; therefore, an extensive examination of the role of disgust toward the political system in conspiratorial tendencies among the Lebanese population is recommended.

## Conclusion

Considering the high level of conspiracy beliefs toward emerging diseases found among the Lebanese population is a vital step to help the country better prepare for the outbreak. Policy-makers should be vigilant and take this phenomenon seriously. Finding ways to reduce people’s reliance on conspiracy theories is, therefore, imperative. Future studies exploring the potential impacts of conspiracy theories on health behaviors were recommended.

## Supplementary Information

**Additional file 1.** Questionnaire MPX

## Data Availability

Data are available from the corresponding authors upon reasonable request.

## Abbreviations

COVID-19: Coronavirus disease 2019
WHO: World Health Organization
MPX: Monkeypox
LGBTQ+: Lesbian, gay, bisexual, transgender, queer, and ally
EVICS: Emerging virus infections conspiracy scale
C.I: Confidence interval
P: *p* Value
M: Mean
SD: Standard deviation
SPSS: Statistical Package for Social Sciences
CVI: Content Validity Index
CVR: Content validity ratio
α: Cronbach’s alpha
CVI: Content validity index (CVI)
I-CVI: Content validity index at the item level
S-CVI: Content validity index at the scale level
