# Supplementary material for: Following the COVID-19 playbook and battling another infodemic: conspiracy beliefs around human monkeypox among the Lebanese population
Source: J Pharm Policy Pract. 2023 Jun 13;16:72. doi: 10.1186/s40545-023-00580-x (PMC10262146; doi:10.1186/s40545-023-00580-x)
Supplement: Supplementary file 1 — Additional file 1. Questionnaire MPX [file 40545_2023_580_MOESM1_ESM.docx]

| **Section1: Sociodemogric characteristics** | |  |  |
| --- | --- | --- | --- |
| **Gender** |  |  |  |
| Male | | | |
| Female |  |  |  |
| **Age** |  |  |  |
| **Marital status** |  |  |  |
| Single |  |  |  |
| Married/Engaged |  |  |  |
| Other (divorced/widowed) |  |  |  |
| **Education level** |  |  |  |
| Secondary or below |  |  |  |
| University or above |  |  |  |
| **Occupation** |  |  |  |
| Outside the medical field |  |  |  |
| Medical field |  |  |  |
| Not working |  |  |  |
| **Urbanicity** |  |  |  |
| Urban |  |  |  |
| Rural |  |  |  |
| **Province** |  |  |  |
| Great Bekaa |  |  |  |
| Great North (Akkar/North) |  |  |  |
| South and Nabatieh |  |  |  |
| Beirut |  |  |  |
| Mount Lebanon |  |  |  |
| **Overall health status** |  |  |  |
| Fair or below |  |  |  |
| Good or above |  |  |  |
| **Religion** |  |  |  |
| Muslim |  |  |  |
| Christian |  |  |  |
| Druz |  |  |  |
| Refuse to answer |  |  |  |
| **Suffering from an immunodeficiency or chronic disease** | |  |  |
| No |  |  |  |
| Yes  **Self-reported economic situation**  Low  Moderate  High |  |  |  |
| **Knowing someone with monkeypox** | |  |  |
| No |  |  |  |
| Yes |  |  |  |
| **Being diagnosed with monkeypox** | | |  |
| No |  |  |  |
| Yes |  |  |  |

**Section 2: Knowledge section: (Response: True/ False/ I don’t know)**

| **General knowledge** |
| --- |
| Human monkeypox is a bacterial disease |
| Monkeypox is a zoonotic viral disease |
| Monkeypox is a gay disease |
| Anyone can be infected by monkeypox |
| Monkeypox is prevalent in Middle eastern countries |
| Monkeypox is prevalent in West and Central Africa |
| Monkeypox is prevalent in Lebanon |
| The incubation period (interval from infection to onset of symptoms) of monkeypox range from 5 to 21 days |
| Period of communicability: 1-2 days before the rash to until all the scabs fall off/gets subsided. |
| **Routes of transmission** |
| Human monkeypox spread easily between people |
| Monkeypox spread faster among gay and bisexual men |
| The virus is a sexually transmitted infection |
| The disease is mostly caught through close physical skin-to-skin contact, which is why it can be spread to sexual partners |
| Human-to-human transmission occurs trough close contact with infectious material from skin lesions of an infected person |
| Transmission can also occur via the placenta from monkeypox mother to fetus |
| Transmission cannot occur via respiratory droplets |
| **High risk groups** |
| Elderly were at high risk of monkeypox |
| Healthcare workers were at high risk of monkeypox |
| **Severity of the diseasse** |
| Monkeypox is less contagious than smallpox and causes less severe illness |
| The clinical manifestation of monkeypox is usually severe |
| Monkeypox is a deadly disease |
| Complications due to monkeypox can occur among children and people with immunodeficiency |
| Complications are related to the extent of virus exposure and patient health status |
| Corneal involvement (may lead to loss of vision) is one of the complication of monkeypox |
| **Clinical symptoms** |
| Monkeypox and smallpox have similar signs and symptoms |
| Monkeypox and chickenpox have similar signs and symptoms |
| Fever, headache, chills, exhaustion, asthenia, back pain and muscle aches are among the early signs of human monkeypox |
| Vomiting and diarrhea are main clinical symptoms of monkeypox |
| Lymphadenopathy (swollen lymph nodes) is one clinical that could be used to differentiate between monkeypox  and other diseases (chickenpox, measles, smallpox) |
| A rash commonly develops one to three days after the onset of fever |
| The rash first appeared usually on the face and spreading to other parts of the body, including hands and feet |
| The cutaneous lesions often first present as macules, evolving to papules, vesicles, pustules, crusts and scabs |
| The number of lesions varies from a few to several thousand |
| Most people infected with monkeypox recover within weeks |
| **Treatment, vaccines and case management** |
| There is no specific treatment for monkeypox |
| Patients should be offered fluids and food to maintain adequate nutritional status. |
| The smallpox vaccine can provide cross-protection for the monkeypox virus |
| There is a specific vaccine for monkeypox |
| The isolation of the monkeypox patient in an isolation room of the hospital/ at home in a separate room is mandatory |
| Covering skin lesions (e.g. long sleeves, long pants) is not necessary for monkeypox cases |
| Isolation should be continued until fever disappear |
| Patients with monkeypox can received antibiotics that alleviate symptoms |
| It is not necessary to closely monitor the monkeypox case during the period of isolation |
| **Precautionary measures** |
| Avoid contact with any materials, such as bedding, that has been in contact with a suspected case of monkeypox |
| Keep safe physical distance |
| Do not touch the rash or scabs of a person with monkeypox |
| Do not kiss, hug, cuddle or have sex with someone with monkeypox |
| Wear a face mask if you are in close contact with someone who has symptoms |
| Eating raw meat |
| Practice good hand hygiene |
| **What to do if you suspect that you (or someone) are infected with monkeypox** |
| If someone catch monkeypox, he should immediately isolate himself from physical contact with others |
| If someone catch monkeypox, there is no need to notify MOPH as the infection is self limited and no need to panic |
| If someone suspect to be infected with monkeypox, he has to seek medical care immediately with giving prior information to the hospital/physician of a potential infection with monkeypox |
| Monitor your symptoms for 21 days since the date of your exposure |
| Notify your contacts that you have been exposed to monkeypox |

| **Sources of information** |
| --- |
| TV |
| Newspaper |
| Relatives |
| Healthcare professionals |
| Health websites |
| Social media |
| Radio |
| Scientific articles |

**Section 3: Attitudes**

**Conspiracy beliefs**

|  | Disagree | Neutral | Agree |
| --- | --- | --- | --- |
|  |  |  |  |
| I am skeptical about the official explanation regarding the cause of monkeypox virus emergence”, |  |  |  |
| I do not trust the information about the monkeypox virus from scientific experts |  |  |  |
| Most viruses including monkeypox are planned and man-made (NIT,simulation exercise) |  |  |  |
| The spread of monkeypoxviruses is a deliberate attempt to reduce the size of the global population”, |  |  |  |
| The spread of monkeypox virus is a deliberate attempt by authorities to gain political control” |  |  |  |
| The spread of monkey virus is a deliberate attempt by global companies to take control including pharmaceutical companies manufacturing vaccines |  |  |  |
| The spread of monkey virus is a deliberate attempt to attack Africain people and to enhance discrimination |  |  |  |
| The spread of monkey virus is a deliberate disctrimatory attempt to attack LGBTQ+ |  |  |  |
| The control measures in response to emerging infection are aimed for mass surveillance and to control every aspect of our lives”, |  |  |  |
| The control measures in response to emerging infection are aimed for mass surveillance and to destabilize the economy for financial gain”, |  |  |  |
| The control measures including lockdown is a way to terrify, isolate, and demoralize a society as a whole in order to reshape society to fit specific interests”, |  |  |  |
| Viruses including monkeypox are biological weapons manufactured by the superpowers to take global control”, |  |  |  |
| Monkeypox was a plot by globalists to destroy religion by banning gatherings”, |  |  |  |
| The mainstream media is deliberately feeding us misinformation about the monkeypox virus and lockdown” |  |  |  |
| The coronavirus disease 2019 (COVID-19) vaccination is linked to the monkeypox outbreak, |  |  |  |
| Microsoft co-founder and billionaire Bill Gates has a role in the outbreak |  |  |  |

|  | Disagree | Neutral | Agree |
| --- | --- | --- | --- |
|  |  |  |  |
| **Attitudes toward precautionary measures** |  |  |  |
| I think that isolation is an effective measure to prevent the spread of monkeypox |  |  |  |
| I think that regular hand hygiene, physical distancing and facemask use could protect people from catchig monkeypox |  |  |  |
| Keeping up with the information regarding the government’s call for MPX preventive efforts is important for the community |  |  |  |
| People with COVID-19 who isolate themselves show that they have a responsibility in preventing the transmission of COVID-19 |  |  |  |

| **Attitudes toward government and health authorities** |  |  |  |
| --- | --- | --- | --- |
| I am confident that the MOPH and local population can control the monkeypox locally |  |  |  |
| I think that Lebanon has adequately scale up the preparedness and response plan for monkeypox |  |  |  |
| I think that there are currently sufficient and adequate prevention and control measures adopted by the health authorities for monkeypox at point of entry |  |  |  |
| I think the response in Lebanon has been sluggish and timid |  |  |  |
| I think that surveillance is spotty and official case counts are likely a gross underestimate. |  |  |  |
| I think that longstanding weaknesses in the public health system will give the virus a chance to become entrenched. |  |  |  |
| I think that monkeypox can add a new burden on the Lebanese healthcare system |  |  |  |
| I think that Lebanon in its current economic situation will be unable to cope with an additional crisis |  |  |  |
| I think that employees strike will delay the early detection and follow up of monkeypox cases |  |  |  |
| I think monkeypox is currently spreading in Lebanon and the real number is likely to be much higher. |  |  |  |
| I think that people need to learn more about monkeypox and the epidemiology of the new emerging diseases |  |  |  |
| I think that it is dangerous to travel to the country's epidemic with monkeypox |  |  |  |
| I think that MOPH have the technical ressources testing that allow her to rapidly detect and glean the extent of the outbreak |  |  |  |
| I think the obstacles to preparedness are systemic, at every level of government |  |  |  |
| I think that MOPH has raised awareness of the disease and its risk factors |  |  |  |
| I think that MOPH have well informed people about the preventive measures they can take to reduce exposure |  |  |  |
